# Supplementary material for: Are carbon emissions trading and green financial instruments synergistic? -Comprehensive quantitative research based on content analysis
Source: PLoS One. 2024 Mar 7;19(3):e0298601. doi: 10.1371/journal.pone.0298601 (PMC10919601; doi:10.1371/journal.pone.0298601)
Supplement: S1 Data — (ZIP) [file pone.0298601.s001.zip › Data/地级市Z_F工作报告.zip!/╜¡╬≈/─╧▓2╩╨/└┤╫╘╣1⁄2╓┌║┼í╛┬φ┐╦╩2╛▌═°í┐_2007.docx]

各位代表：

现在，我代表南昌市人民政府，向市第十三届人民代表大会第一次会议报告过去五年的工作，对今后五年的工作构想提出建议，请予审议，并请各位市政协委员和列席会议的同志提出意见。

一、过去五年工作回顾

2001年以来,在省委、省政府和市委的正确领导下，全市人民坚持以邓小平理论和“三个代表”重要思想为指导，全面落实科学发展观，紧紧围绕富民强市目标，解放思想，善谋实干，全力做好建设现代区域经济中心城市和现代文明花园英雄城市“两篇文章”，圆满完成了市第十二届人民代表大会确定的各项目标任务，全市国民经济和社会发展取得了巨大成就，上了一个历史性的新台阶，为实现南昌崛起的新跨越打下了坚实基础。

（一）经济总量实现五年翻番，现代区域经济中心城市地位初步确立

初步预测，2006年全市实现地区生产总值1185亿元，比2001年增长1.07倍（以下简称同比）；财政总收入150亿元，同比增长1.8倍；地方一般预算收入65.3亿元，同比增长1.9倍；全社会固定资产投资683亿元，同比增长6.1倍；社会消费品零售总额358.5亿元，同比增长99.2%。三大产业比重由10.2:45.9:43.9调整为6.4:54.6:39.0。“十五”期末，我市十二项主要经济指标中，有五项增幅名列全国省会城市前五位，综合实力由全国百强城市第58位上升到第35位。

1、开放型经济取得突破性进展。2001—2005年累计，实际利用外资27亿美元，年均增长94.2%；实际利用内资684亿元，年均增长53.5%。已有768家外资企业、2030家内资企业落户南昌，其中世界500强20家、国内200强22家。预计2006年实际利用外资11亿美元，实际利用内资305亿元，出口创汇12亿美元。今年6月，南昌以投资的活力、发展的速度和日益增长的后劲，被美国《新闻周刊》评为“世界十大动感都会”之一。全民创业势头强劲，预计到2006年底，全市个私民营企业总数达到13.1万户，年度个私民营经济纳税额37.4亿元，比上年增长23%。

2、先进制造业基地建设成效显著。2001—2005年，全市规模以上工业增加值年均增长25%，预计2006年完成304亿元，较上年增长26%。南昌高新技术产业开发区2006年主营业务收入突破300亿元、财政收入过10亿元，成为国家科技兴贸出口创新基地；南昌经济技术开发区跻身中西部地区同类开发区前三甲；南昌县小蓝经济开发区等工业园区形成了各具特色的发展格局。目前，全市已形成汽车、医药和食品、电子信息和家电、纺织服装、冶金和新材料五大支柱产业。拥有中国名牌产品6个，中国驰名商标6件，国家免检产品18个，江西省名牌59个，江西省著名商标68件。

3、农业基础进一步巩固。认真执行各项支农、惠农政策，实施农村税费改革和取消农业税，国家粮食补贴政策执行到位。建成了特种水产、花卉苗木、无公害蔬菜等8大特色种养示范基地，拥有市级以上农业产业化龙头企业95家。除险加固病险水库，修建山塘、门塘，检修机电泵站，硬化堤顶路面，农业基础设施进一步夯实。大力推广农业科技，粮食作物优质品率、生猪良种率、水产品优质品率明显提高。预计2006年粮食播种面积510.8万亩，总产量203.15万吨；禽蛋产量12.1万吨，家禽出笼3397万羽，生猪出栏237万头。

4、商贸服务业迅猛发展。商贸业初步形成城内大商场、城中特色街、城郊大市场、城外大物流的格局。已有特色街9条、超万平方米商场17个、10亿元以上交易额的市场6个。旅游业发展较快，2001—2005年，旅游总收入183.5亿元，预计2006年实现旅游总收入55亿元，较上年增长18.8%。会展经济异军突起，2002—2006年，成功举办五届金秋经贸活动月，来昌客商4万余人，引进项目700多个。2006年被评为中国十大节庆城市之一，绳金塔庙会文化经贸节荣获“中国十大经贸类节庆奖”，中国南昌傩文化艺术旅游节荣获“中国十大民俗类节庆奖”。强力打造政银企合作平台，与各大商业银行开展信贷合作，上海浦发银行、福建兴业银行相继落户。2006年，南昌被评为中国十佳金融生态城市之一。

（二）城乡面貌全面改观，现代文明花园英雄城市魅力日益显现

1、城市框架迅速拉开。按照“一江两岸，南北两城，双核拥江，外带五个组团”格局，2001—2005年，全社会城市建设投入250亿元，城市面貌发生了巨大变化。东、西外环2条高速公路建成，红谷滩滨江新城快速推进，高新技术开发区、经济技术开发区迅速拓展，瑶湖、前湖两个高校园区建设成形。南昌城区面积由2001年85平方公里扩大到2006年210平方公里。

2、城市功能不断增强。加强防洪体系建设。全市10万亩以上滨湖圩堤防洪标准由10年一遇提高到20年一遇，昌南防洪标准达到100年一遇。改善城市道路交通。修建一级公路3条，打通出城道路15条，改造城中道路68条，整治小街巷485条，生米大桥竣工通车，洪都大桥开工建设。初步形成了环形放射状城市快速干道网框架。启动昌北国际机场改造，建成洪城、昌南两个大型综合客运站和徐坊长途客运站；新增公交车线路103条，基本满足了市民日常出行需求。提升城市供排水能力。完成了青云水厂三期，新建了长堎水厂、牛行水厂、扬子洲水厂，启动了红谷滩、红角洲、凤凰洲供水工程，城市供水体系覆盖面进一步扩大；实施12大项30个子项涉水工程建设，基本解决城区积水问题。完善城市电网系统。新建了110千伏、220千伏、500千伏变电站20座，着力改造老城区电网，实现了“寒冬不拉闸、酷暑不限电”。强化服务平台搭建。开通南昌—深圳、南昌—厦门、南昌—香港海铁联运，建设南昌出口加工区，启用国际集装箱码头，实施火车站西广场改造。

3、城市管理水平提高。按照“财权与事权统一”原则，合理调整了东湖、西湖、青云谱、青山湖四个城区的区划，下移城市管理重心，形成城市管理综合执法体系。以创建“全国文明城市、国家卫生城市、国家环保模范城市、全国园林城市”为抓手，实施文明南昌行动计划，推进文明行风、文明表率、文明市民、文明村镇、文明社区五大工程，市容市貌发生根本变化，城市文明程度和市民文明素质显著提高。2005年，被评为创建全国文明城市工作先进市。特别是卫生城市创建，历经十六年风雨艰辛，今年终于获得了“国家卫生城市”殊荣。

4、城市品位明显提升。按照“历史名城，山水都城，现代新城”的要求，挖掘历史文化底蕴，弘扬革命传统精神，绿化亮化美化城市。改造后的八一广场，更加庄严雄伟；建成后的秋水广场、阳明公园、朝阳公园、赣江市民公园等，成为南昌城市新名片；整治后的两河八湖，彰显了南昌“水城”特色。城市绿化覆盖率达38.6%；路灯、景观灯达12万盏；空气质量优良率达92.8%，生活污水处理率达71.7%，生活垃圾处理率达100%。2006年，荣获“中国人居环境奖——水环境治理优秀范例城市”称号。

5、新农村建设有序推进。在加快农业经济工作的同时，推进社会主义新农村建设，开展了“百乡动员、千村示范、万村整治、百万人行动”的农村环境卫生整治活动，实施了以“三清三改”为主要内容的村庄整治，突出1000个自然村的整治试点,全面实施农村学校危房、敬老院、卫生院改扩建工程，实现行政村公路全面硬化。目前，全市已有6个镇被建设部列为国家重点镇，14个镇被列为省级重点镇，28个村镇荣获“省级文明村镇”称号；南昌县蒋巷镇、塔城乡北洲村、进贤县文港镇等荣获“国家级文明村镇”称号，进贤县李渡镇被评为“全国新农村建设示范镇”；青山湖区进顺村被评为2004年“全国十佳小康村”、2005年“中国十大名村”和“全国文明村”；青山湖区湖坊村被评为2005年“全国十佳小康村”。

（三）社会事业全面进步，城乡人民生活明显改善

1、大力发展各项社会事业。教育事业稳步发展。均衡发展基础教育，提升发展高等教育，大力发展职业教育，基本消灭中小学危房。小学、初中入学率分别为99.99%和99.7%。职业教育进入全国“前三强”。科技兴市成效显著。国家半导体照明工程产业化基地落户南昌，成功加入世界科技城市同盟，荣获2003—2004年度全国科技进步先进市、全国知识产权示范创建城市。2001—2005年，全市荣获国家级、省级科技进步奖442项。人才强市格局基本形成。实施人才绿卡、优秀人才特殊引进、高层次人才安家补贴、高层次人才生活津贴、引进人才奖励等制度，目前全市拥有42.86万名各类人才，实现了人才流入大于流出的突破。卫生事业成绩突显。推进市疾病控制中心和南昌紧急救援中心建设，加强疾病预防控制，实现了“非典”和“人禽流感”疫情零的记录；强化基层卫生机构建设，建成城区社区卫生服务站226个，城市二级医疗预防保健网络基本形成；探索血吸虫病防治新模式，农村新型合作医疗试点受益面达150余万人，农村三级卫生服务网络基本建立。文化广播事业彰显魅力。文化艺术精品获国家金奖9枚，省级金奖88枚；广播剧《马克思的一天》获中宣部“五个一工程”创作奖。荣获首座全国楹联文化省会城市荣誉称号。启动八一起义纪念馆改造，新建南昌市博物馆。推进城区有线电视数字化试点改造，大力发展农村有线电视，实施广播电视“村村通”工程，全市广播电视综合覆盖率达97%。体育事业硕果累累，荣获世界冠军10个，亚洲冠军9个，全国冠军38个；全民健身运动广泛普及。

2、不断提高人民生活水平。2001—2005年，新增城镇从业人员42.16万人，向省外劳务输出104.2万人次。全市养老保险参保61.5万人，失业保险52.67万人，基本医疗保险37万人，工伤保险53.41万人，生育保险37.41万人。统筹范围内的离退休人员养老金100%按时足额发放，城市特困低保户做到了应保尽保，低保标准城区由143元提高到210元、县区由104元提高到170元。2001—2005年，共下拨农村特困户救助资金3591万元，五保户供养资金1.5亿元。住房条件改善，全市城镇新建房屋竣工面积1200万平方米，经济适用房面积94万平方米；对1.3万户困难家庭实行了公有住房租金核减,对2428户“双困”家庭实行了廉租房租金配租和实物配租。预计城市居民人均可支配收入由2001年6206元上升到2006年11228元，农民年人均纯收入由2001年2517元上升到2006年4286元。

3、稳步推进依法治市工作。完成机构改革任务。各级政府部门精简28.6%,市县乡镇人员总体精简32%。加强政府法制工作。2001—2006年，提请市人大常委会审议的地方性法规草案17件，制定规章22件，修改规章41件，废止规章88件；推行行政执法责任制和行政首长问责制，严格追究执法过错责任。受理行政复议案件556件。严格落实《行政许可法》，取消行政审批事项694项，保留的行政审批事项为全国最少的城市之一。重视法制宣传教育，荣获全国2001—2005年“四五”法制宣传教育先进城市称号。开展“投资环境年”、“机关作风年”、“机关效能年”、“双百评”及“服务行业专项评”活动，改进了机关作风，提高了行政效能，优化了投资环境。强化社会治安综合治理，认真做好信访工作，全力维护社会稳定。五年来，全市没有发生大规模群体事件、严重暴力性事件、重大治安事件、恶性火灾事件、重特大生产经营性事故，被评为2001—2004年度全国社会治安综合治理工作先进市。政府各部门、各执法单位能够文明、公正执行公务，取得明显成效。

认真落实人口、资源、环境三大基本国策，完成计划生育指标任务，重视生态环境保护，强化土地资源管理。开展国防教育，做好双拥工作，加强人防建设，荣获全国双拥工作“五连冠”、全国人民防空先进城市称号。外事侨务、民族宗教、对台事务工作继续加强，统计、审计、参事、档案、地方志、保密、老龄、工会、共青团、妇女、儿童、慈善、残疾人、消费者权益保护、防震防灾等工作，取得骄人业绩。

各位代表，上述成绩的取得，是全市人民在市委的正确领导下，认真贯彻党中央、国务院和省委、省政府的大政方针，团结奋斗、扎实工作的结果；是市人大依法监督和市政协民主监督、支持、参与的结果；是各民主党派、工商联、无党派人士、各人民团体和驻昌单位、人民解放军、武警官兵以及离退休老同志共同努力和大力支持的结果。在此，我谨代表市人民政府，向全市人民，向所有关心、支持南昌发展的同志们、朋友们，表示衷心的感谢并致以崇高的敬意！

回顾过去的五年，我们深深地体会到，南昌的发展必须坚持解放思想，开拓创新，以开放提升城市活力；必须坚持科学发展观，以经济提升城市实力；必须坚持规划、建设、管理并重，以环境提升城市品位；必须坚持以人为本，和谐发展，以文明提升城市形象。这是我们工作的宝贵经验，在今后仍然要加以坚持。同时，我们也清醒地看到前进中存在的困难和问题。主要是：我市仍属欠发达城市，综合实力还不强，经济社会发展总体水平亟待进一步提高；城市承载功能有待进一步完善，管理水平有待进一步提升；农村基础仍然偏弱，社会就业压力较大；资源节约和环境保护任务较重；政府效能仍需进一步提高。对此，我们务必采取切实措施，认真加以解决。

二、今后五年构想

今后五年，是做好“两篇文章”、加速富民强市的重要五年，也是完成“十一五”规划、构建和谐文明南昌的关键五年。2005年，我市人均地区生产总值2.2万元；预计今年，将达2.6万元，跨上人均地区生产总值3000美元的新台阶。纵观世界各国的经济发展历程，这个阶段的突出特征是：产业结构由低层次向高层次发展，一、二产并重向二、三产并重发展，劳动密集型向资本密集型、技术密集型发展；消费层次由低层次向高层次升级，基本衣食住行消费向高级衣食住行和文化旅游消费发展。站在这一新的历史平台，机遇与挑战并存、希望和困难同在。一方面，经济全球化趋势深入发展，知识网络化激流奔腾而来，科学技术扩散日益加快，国际产业分工纵深发展，给我们带来了十分有利的发展条件；尤其是国家支持中部崛起战略的全面实施，省委、省政府支持南昌做强做大，强化南昌作为全省发展核心增长极作用，把南昌建成我国中部地区重要的先进制造业重要基地和商贸、物流、金融中心，构建以南昌为核心的环鄱阳湖城市群的战略决策，为南昌提供了更为广阔的发展空间；另一方面，国际贸易保护主义加剧，能源、原材料价格上涨，部分产业产能过剩，国家加强土地、资金宏观调控，城市间竞争日益加剧，又对我们的工作提出了新的更高要求。面对新的发展形势，站在新的历史平台，南昌要在中部地区快速崛起，就必须抢抓机遇，乘势而上，进一步解放思想，以更加开阔的视野、更加有力的举措、更加务实的作风，不断开创南昌经济社会又好又快发展的新局面。

今后五年政府工作的总体构想是：高举邓小平理论和“三个代表”重要思想伟大旗帜，坚持以科学发展观统领经济社会发展全局，按照市第九次党代会确定的发展思路，围绕“一个基地、三个中心、四个南昌”的总要求，坚定不移地实施大开放主战略、新型工业化核心战略、城乡一体化战略、科教兴市战略和可持续发展战略，全面提升产业、人口、资源的聚集和辐射能力，不断增强南昌的综合实力和核心竞争力，促进社会和谐，加快富民强市，把南昌基本建设成为现代区域经济中心城市和现代文明花园英雄城市。

今后五年全市经济主要预期目标：

2011年地区生产总值达到2600亿元，力争达到2800亿元；

2011年财政总收入达到300亿元，力争达到350亿元；全社会固定资产投资年均增长23%，力争达到25%；

社会消费品零售总额年均增长15%，力争达到16%；

城市居民年人均可支配收入年均增长9%，力争达到10%；

农民年人均纯收入年均增长8%，力争达到10%；

单位生产总值能源消耗比2006年下降20%，

人口自然增长率控制在8‰以内。

为了实现上述要求和目标，必须扎扎实实地深入推进以下战略举措：

（一）坚定不移地实施大开放主战略，着力营造全国最适宜投资创业的发展环境

以打响“全球十大动感都会”城市品牌为契机，进一步提高开放型经济发展水平，认真探索更高层次开放和更高效益开放的新途径，加快把南昌建成中部地区承接国际性产业资本转移和国内产业退沿进中的重要基地。

1、大力提升招商引资质量。充分利用南昌资源、生态、区位等要素优势和现有产业基础，对接长珠闽、联结港澳台、融入全球化，策应“万商西进”工程，利用“9+2”泛珠三角区域经济协作平台，加快招商引资向招商选资转变步伐，注重承接产业梯度转移、高端产业直接转移，突出吸引战略投资者、重大项目先进技术和高素质人才。重点引进符合我市产业规划与整体发展战略的跨国公司、国内大型企业特别是世界500强、国内200强、港澳台500强、地区性总部、研发中心、结算中心、营运中心和采购中心，力争使实际利用内外资在今后5年内有较大幅度增长，在中部省会位居前列。

2、大力发展外贸外经。优化外经贸发展结构，扩大机电产品、高新技术产品出口份额，加快建立光机电一体化、电子信息、汽车、服装、农产品出口产业基地；积极引导和支持有条件的企业进入世界500强企业采购体系，直接带动产品出口。实施科技兴贸战略，转变外贸增长方式，提高高新技术产品出口创汇比重，力争出口创汇实现跨越式发展。推动有条件的企业“走出去”，加快融入全球产业链、营销链和供应链；拓展国际工程承包，带动国际劳务合作和建筑材料出口；加快发展服务外包业务，尽快使我市成为全国服务外包重要基地。

3、大力营造良好投资环境。牢固树立“五为”理念，深入打造“四最”品牌，不断创新招商、亲商、安商、富商方式，重点营造“四大”开放优势，即建立和完善与国际惯例和沿海城市对接的政策法规体系，营造开放的制度优势；加强国际间友好城市的协作与交流，加强国内区域合作，实现市场、设施、生态对接，做到要素互补、资源共享、利益互惠，营造开放的协作优势；积极推进大通关工程，以海铁联运为突破口，降低进出口货物运输成本，建设快捷、便利的进出口通道，完善海铁联运体系，加快出口加工区建设，启动保税物流中心，营造开放的成本优势；加快电子口岸建设，加强行政服务中心和招投标中心等利企便民的政务平台建设，实现一站式网上办事服务，营造开放的服务优势。

（二）大力转变经济增长方式，着力壮大先进制造业重要基地

策应环鄱阳湖城市群核心城市和中部地区区域经济中心城市建设进程，进一步突出工业经济的主导地位，加快产业集聚，走新型工业化的路子。坚持以开发区为载体，优先发展高新技术产业，以信息化带动工业化，以工业化促进信息化。加快用高新技术改造提升传统产业，做到传统产业升级与新兴产业壮大并重、加工制造业与装备制造业并重，在更大范围、更宽领域、更高层次上参与国际、国内分工协作，努力使南昌成为中部地区先进制造业重要基地。

1、强力实施重大项目，培植优强企业和产业集群。倾力支持驻市企业做强做大，大力扶持市属骨干企业加快发展，全力帮助新上项目早开工、早投产、早达标、早见效。以实施重大项目为突破口，建立“3010”工程目标考核体系和工作推进机制，明确目标，分解任务，通过引进联合，重点投资建设10个30亿元和30个10亿元市场前景好、技术含量高、产业关联度大、带动作用强的重大项目。坚持市领导挂点、部门联动的服务重点企业的工作机制，切实帮助企业解决困难和问题。力争到2011年，新增3家以上年销售收入过100亿元、5家以上年销售收入达50亿元的骨干企业。围绕汽车、电子信息、空调、机电产品、医药及医疗器械、食品、造纸、飞机、冶金及新材料、纺织服装十大产品制造基地核心企业，开展专业化协作和产业配套，延伸产业链，催生企业簇群，形成具有较强集聚和辐射效应的产业集群。

2、强力优化功能布局，精心打造特色园区。建立统一协调全市工业园区发展的工作机制，进一步明确园区的产业定位，优化功能布局，引导园区集约发展、错位发展，使工业园区成为产业特色明、创新能力强、投资回报高的制造中心和产业基地。南昌高新技术产业开发区重点发展电子信息、生物医药、光机电一体化、新材料、应用软件等；南昌经济技术开发区重点发展微电子、汽车、空调等；红谷滩新区重点发展生物高科技产业等；小蓝经济开发区重点发展汽车及零部件制造业等；桑海开发区重点发展生物医药等；英雄开发区重点发展电子机械、奶制品业等。

3、强力支持科技创新，加快发展高新产业。坚持走“引进、消化、吸收、再创新”的路子，大力发展高新技术产业，采用高新技术和先进适用技术改造提升传统产业，推动高新技术产业化和产业高新技术化。加快建设企业为主体、市场为导向、产学研相结合的技术创新体系。鼓励企业自主研发，支持有条件的企业建立博士后工作站和研发中心。进一步强化激励措施，加大支持力度，建立和完善风险投资机制，鼓励自主创新，逐步变“南昌制造”为“南昌创造”。到2011年，力争培育国家级企业技术中心5家以上，博士后工作站10家以上，建立以国家级开发区、留学生创业园、大学科技园、金庐软件园等为创新载体的孵化器20家以上，努力把南昌建成全国科技进步示范市和中部地区高新技术产业化基地。

4、强力推动品牌战略，不断增强竞争能力。鼓励和支持企业实施品牌经营，加大对本地现有中国驰名商标和中国名牌产品的保护扶持力度，重点培育一批年销售收入过亿元、市场占有率进入全国同行业前十名的精品名牌。力争到2011年，新增国家级品牌10个以上、省级品牌100个以上。大力发展循环经济，积极推广节能技术，鼓励发展节能产品，加大对重点耗能企业的监控力度。全面推行清洁生产，加强环境保护，从源头上减少废物排放，切实提高资源综合利用率。

（三）大力发展现代服务业，着力形成中部区域性商贸、物流、金融中心

朝着把南昌建设成为中部集聚资本、辐射商品贸易的现代区域性服务中心城市目标，优先发展现代物流业、商务服务业、软件业、金融服务业等与先进制造业配套的生产性服务业，大力培育带动力强的旅游业、信息服务业、会展业等新兴服务业，提升商贸业、社区服务业等传统服务业，推动先进制造业与现代服务业相互促进、共同发展。

1、大力提升传统商贸业。完善网点规划，优化网点布局，构建分工明确的商业层级服务体系。提升老城区市级商业中心，建设红谷滩中央商务区，打造城东、城南商业副中心，加快区级商业中心、卫星城镇商业中心、社区商业网点和专业特色街建设步伐，推进“万村千乡”市场工程的农家店建设，在城郊结合部发展一批大型规范的专业市场，初步形成多层次、多类型的商业服务体系。支持现有商贸龙头企业发展连锁经营，吸引国内外知名商贸龙头企业进驻南昌。到2011年，力争年销售额5亿元以上的商贸企业达到20家，使南昌成为各类商品和服务贸易的重要集散地。

2、大力发展现代物流业。以降低物流成本、提高经济运行质量为目的，突出与口岸、专业市场、产业园区相配套的物流设施建设，重点建设以向塘编组站、青云谱货场和三条国道为轴心的铁路——公路货运枢纽型物流园区，以昌北国际机场、南昌港以及国家级保税仓库、保税物流中心、出口加工区为依托，以海铁联运、河海联运为纽带的进出口贸易型物流体系；吸引、鼓励和支持物流企业做强做大，提升现代物流层次和水平。

3、大力发展金融保险业。充分发挥金融业在市场资源配置中的核心作用，继续支持各商业银行发展，在防范风险的前提下，鼓励金融机构扩大信贷规模，提高信贷质量，提供优质服务；吸引金融、保险等生产性服务业进驻南昌，五年内力争引进全国性股份制商业银行分支机构5家，外资银行分支机构2家；大力发展商业人寿保险和个人储蓄性养老保险；支持企业上市，提高上市公司再融资能力。

4、大力发展旅游会展业。着眼把南昌建成面向海内外旅游休闲的后花园，突出红色摇篮、绿色家园主题，重点整合南昌的红色文化、历史文化、民俗文化等资源，增强旅游的文化内涵，打造自然景观与人文景观相互融合、特色鲜明的精品旅游线路，吸引更多的国内外游客观光南昌；依托全省丰富的旅游资源，充分发挥南昌交通、区位和接待优势，加大区域性旅游合作，尽快使南昌成为区域性旅游集散中心。力争旅游总收入年均增长18%以上，其中旅游创汇年均增长25%。继续发展会展经济，积极承接国际国内大型会展，培育南昌品牌展会，进一步发挥会展的经济助推器作用。

5、稳步发展房地产业。加强政府宏观调控，建立以中低收入家庭为主的住房保障体系，建立满足高中低档不同需求的住房供应体系，形成“供需基本平衡、结构基本合理、价格基本稳定”的发展格局，全面提高人民群众的居住质量。科学制定年度住宅开发计划，稳定商品房价格，使中小户型普通商品房、经济适用房和廉租房建设量占住房建设总量的70%。

（四）以承办全国第七届城市运动会为契机，着力打造功能更加完善、管理更加科学的魅力都市

2011年，我市将承办第七届全国城市运动会，这既是展示我市经济社会发展成就的难得机遇，也是对我市城市规划、建设和管理水平的全面检验。我们要以此为契机，坚持高起点规划、高质量建设、高水平管理，全面展示南昌的独特魅力和现代风貌。

1、优化城市空间布局。按照国务院批准的总体规划要求，策应环鄱阳湖城市群的构建，重点深化城市发展战略性规划，开展编制产业发展布局等一系列专项规划，启动沿江、沿湖地区城市设计；加快与周边城市基础设施和区域功能的相互衔接。积极推进城市四大功能区的规划和建设，以老城为主的提升区、以新城和开发区为主的功能优化区、以外围城镇为主的一般拓展区、以山水湿地风景名胜为主的生态保护区。推进老城区城中村、低洼危旧房等改造，降低老城区人口密度，优化老城区功能；推进朝阳地区基础设施建设和开发，提升红谷滩中央商务区功能，加强南昌水城的特色风貌研究；完善开发区的配套功能，提高开发区的集约利用水平。

2、完善城市承载功能。以路、水、电、气为重点，抓好八大工程建设。一是城市畅通工程。完善绕城高速公路网，建设昌进一级公路二期工程，拓宽昌万公路，形成“半小时经济圈”骨架公路网。加快“三环十一射”道路建设，抓紧洪都大桥、火车站广场改造及打通洛阳路等重点工程建设，启动昌九城际铁路、南昌铁路西客站、白水湖铁路货运枢纽站等项目建设，规划建设城市轨道交通，完善老城区快速交通干道系统，科学组织交通微循环，提高现有道路通行能力。扩建昌北国际机场，使之成为通航国内外重要城市的中型枢纽港。大力实施“公交优先”战略，发展城市公共交通，完善城市交通基础设施，加强交通管理，开展疏堵治乱工作，有效缓解城市交通拥堵问题。二是城市供排水工程。筹建城北水厂、红角洲水厂、幸福水厂，扩建城南水厂，逐步形成东至瑶湖、西到湾里、南至莲塘、北到乐化的区域供水体系。解决朝阳地区排水不畅问题，分期分片建设与改造污水处理厂和下水道支管，老城区由雨污合流逐步转变为雨污分流，新城区采用雨污分流制。适时提高污水处理能力，使污水集中处理率进入全国先进行列。三是城市电源电网工程。力争新建新昌电厂，扩建南昌燃气电厂，进一步增强配电网整体供电能力，确保安全稳定供电。四是城市气网工程。大力发展燃气配套设施，提高管网气化率；进一步完善城市燃气管网，加快引进天然气，形成合理的气源结构。五是城市景观工程。积极支持梅岭生态环境的保护建设，加大艾溪湖等六大风景区、红角洲南昌水城等建设力度，继续抓好玉带河、乌沙河、桃花河、五干渠等景观风光带建设，加快“一江穿城、西山为屏、多湖相拥、绿廊成网”的城市绿地系统构建，创建国家生态园林城市。挖掘文化底蕴，保护历史名胜、革命旧址、人文遗址、民俗景观，提升城市品位。六是“数字南昌”工程。构筑通讯网络、计算机网络、有线电视网络三网融合的城域网络，建设电子政务服务网络体系框架，搭建电子商务公共服务体系平台，实施城市综合服务体系重点应用项目。七是城市防洪工程。加快城北防洪工程建设，防洪标准提高到100年一遇；县区城防标准提高到50年一遇。八是防灾减灾工程。完善气象、地震等灾害监测预报预警体系，加强人工影响天气工程、防治生物灾害综合体系建设，实施地质灾害避让、搬迁工程和平战结合的人防工程，不断提升南昌的防灾减灾能力。

3、实施科学长效管理。以跻身“国家卫生城市”为新的起点，以创建全国文明城、国家环保模范城、国家园林城为抓手，继续完善“两级政府、三级管理、六级网络”城市管理体制，按照建管同步、建管并重、依法管理、科学管理原则，做到条块结合、以块为主，重心下调，责权利相统一，事权财权相统一。加强城市管理立法，建立健全城市管理目标责任制，制定符合市情的城市管理标准化体系，实现城市管理的信息化、网络化、精细化、长效化。继续实施“文明南昌行动计划”，深入推进“五大文明工程”，全面提升城市管理水平。

（五）大力发展县域经济，着力建设社会主义都市新农村

围绕社会主义新农村建设目标，坚持统筹城乡发展，贯彻“工业反哺农业、城市支持农村”和“多予、少取、放活”方针，继续加大农业投入和扶贫、扶优、扶乡力度，发展县域经济，夯实农村基础，促进城乡融合，促进农业增效、农民增收、农村发展。

1、全力发展县域经济。坚持走工业化、城镇化和农业产业化协调发展的路子，突出产业特色，形成产业集群。民营科技园、长堎工业园、昌东工业园区、昌南工业园、大温圳工业圈、安义工业园、湾里罗亭工业园等必须依托优势、准确定位、错位发展、形成特色。力争到2011年，有一两个县区跨入全国百强县区行列。立足把南昌建成全国优质农副产品生产加工基地，坚持以市场为导向，按照品种特色化、基地规模化、生产标准化、经营产业化、投入科学化的要求，严格保护基本农田，稳定和提高粮食综合生产能力；加快农村合作经济组织发展步伐，积极推进“一村一品”工程；大力支持农业产业化龙头企业扩张，发展特色农产品、无公害农产品、绿色食品和有机食品，进一步提高农产品附加值和质量安全水平。力争到2011年，市级以上龙头企业超过100家。

2、全力促进农民增收。进一步落实各项支农、惠农政策，切实加强农民负担监管力度，维护农民合法权益；加大财政扶持力度，深入推进扶贫开发，支持改善农村生产和生活条件；继续实施村党支部书记“双带”致富工程，推动农民创业致富；继续实施农民转移就业工程和农民工培训“阳光工程”，增加农民劳务收入；加强和完善农村社会保障制度，为农民排忧解难。

3、全力建设都市新农村。坚持统筹城乡规划建设，着力抓好小城镇建设，重点建设向塘、李渡、鼎湖、生米、扬子洲、梅岭6个改革与发展试点镇和向塘、蒋巷、三江、西山、望城、李渡、文港、温圳、万埠、罗亭、麻丘11个农村建设示范镇。消灭“空壳村”，合理撤并小型村，做大中心村。进一步加快村庄整治步伐，每年抓好一批试点自然村整治，让农民走平坦路、喝干净水、上卫生厕、用节能灶、洗舒适澡、过安稳日子。加强农村基础设施建设，重点加快农村公路、植树造林、水利工程、学校危房改造、敬老院、卫生院“六大工程”建设步伐。在实现行政村道路硬化的基础上，力争到2011年，全市农村自然村道路硬化率达到75%；加大投入力度，提高圩堤防洪排涝标准，完成病险水库除险加固；农村自来水普及率、沼气入户率、村庄绿化率、农户水冲厕比例、婚育新风入户率、农村商业网点覆盖率、有线电视覆盖率走在全省前列。

（六）坚定不移地深化各项改革，着力创新适应社会主义市场经济的体制机制

实现南昌在中部地区率先崛起，必须进一步深化改革，让劳动、知识、资本、技术、管理的活力竞相迸发，让创造社会财富的源泉充分涌流。

1、基本完成国企改制。按照“归属清晰、权责明确、保护严格、运转流畅”要求，加快建立现代企业产权制度。通过规范上市、中外合资、兼并收购等多种手段，加快国有大中型企业股份制改革，大力发展混合所有制经济。以实现国有资产保值增值和维护职工合法权益为前提，合理调整国有资产布局，积极探索国有资产监管和经营的有效形式。

2、加快投融资体制改革。坚持政府引导、社会参与、市场运作，归集重组城市资源，完善多元化投融资机制。健全以规划为依据，以土地和环保为约束，以产业政策和市场准入条件为导向，与财政、税收、金融等密切配合的投资宏观调控体系，实行便捷有效的项目备案制、核准制和审批制，充分发挥市场配置资源的基础性作用。

3、推进财政体制改革。大力培植财源，做大财政蛋糕。全面完成“乡财乡用县监管”改革。加强依法治税，鼓励城区建立街道财政，支持街道财政所的建设，将城区属地的增值税与城区财政利益挂钩，实行超收分成，调动城区协税护税的积极性。严格资金管理，坚持勤俭节约办事。继续深化部门预算改革，制定部门预算执行绩效考核评价办法，对预算执行和专项资金使用效果进行评价，提高财政资金使用效益。进一步扩大公共财政覆盖面，财政资金更多地向农村倾斜、向社会事业发展的薄弱环节倾斜、向基层倾斜、向困难群体倾斜，让广大人民群众共享改革发展的成果。

4、推进事业单位改革。实行科学分类管理，引入市场竞争机制和社会参与机制，把经营性事业单位进一步推向市场。积极推进文化、教育、广播电视等领域体制改革，抓紧组建市广播电视集团、演艺集团。以转换用人机制和搞活用人制度为核心，加快事业单位人事制度改革，进一步激发事业单位活力。

5、深化农村综合配套改革。以转变政府职能为重点，深化乡镇机构改革；以落实教育政策保障机制为重点，深化农村义务教育改革；以增强基层财政保障能力为重点，深化县乡财政管理体制改革；稳定和完善以家庭承包经营为基础、统分结合的双层经营体制，按照“依法、自愿、有偿”原则，规范流转土地承包经营权，发展多种形式的规模经营；深化农村流通体制改革，提高农产品商品率；按照“明晰产权、减轻税费、放活经营、规范流转”要求，积极抓好林业产权制度改革；按照“成熟一个改制一个”原则，稳妥推进农场经营体制改革。

（七）坚持以人为本，统筹发展，着力构建富裕、和谐、平安的文明南昌

以解决人民群众最关心、最直接、最现实的利益问题为重点，大力发展社会事业，倡导和谐文化，完善社会管理，促进公平正义，促进人与自然、人与人、人与社会和谐相处。

1、高度关注人民生活。继续实施“就业再就业”一号工程，加大农村劳动力转移力度，千方百计增加城镇就业岗位，给有就业需求的“零就业家庭”至少提供一个就业岗位，城镇居民登记失业率控制在3.45%以内。建立健全多层次养老保险、失业保险、医疗保险、工伤保险、生育保险和慈善救助的社会保障体系。加大财政资金投入力度，有效解决住房难、上学难、看病难等与广大群众切身利益密切相关的现实问题。继续加大廉租房建设力度，扩大经济适用房建设规模，逐步解决困难群体的住房问题。

2、深入推动全民创业。大力营造创业光荣的氛围，进一步引导形成经济社会发展的内生机制；完善创业政策，搭建创业平台，激活创业主体，鼓励城乡各类人群投身创业；丰富完善中小企业公共服务平台功能，加速中小企业创业基地建设，突破创业融资难瓶颈，扶助创新型企业成长；倡导诚信创业，推动全民创业向纵深发展，使南昌成为最适宜创业的城市。

3、优先发展教育事业。围绕打响“学在南昌”品牌，加大教育事业的投入，新增教育经费重点支持农村和薄弱学校的建设，在全市免除义务教育学杂费，全面普及义务教育。均衡发展基础教育，重视学龄前幼儿教育和特殊教育，抓好市区教育网点布局调整，逐步解决老城区校园面积过小、新城区学校网点不足的问题。调整中等职业学校布局和结构，推动高等职业教育发展，把南昌建设成为全国重要的职业教育中心。

4、大力发展卫生事业。围绕建设“健康南昌”，坚持政府主导，强化公共卫生体系建设，加快农村卫生和社区卫生发展，全面推进新型农村合作医疗，完善城乡医疗救助制度，建立“分级保障、低水平、广覆盖、大病救助、特殊人群救助”的全民医疗保障体系，为群众提供安全、有效、方便、价廉的公共卫生和基本医疗服务。统一规划管理区域内的医疗机构和卫生资源，鼓励和引导社会资源发展卫生事业，大力支持民营医院发展，促进多渠道办医格局形成。

5、繁荣活跃先进文化。大力倡导和谐文化，建设公益性文化设施，发展文化产业，打响历史名城名片。以创建全国文明城为契机，积极开展社会主义荣辱观教育，把社会和谐建立在共同的理想和价值取向上。以建军80周年为契机，加快推进八一起义纪念馆、新四军军部旧址、八大山人纪念馆等改扩建工程；尽快启动报业大厦、市群众艺术馆、市博物馆二期工程、南昌大剧院建设；合理规划布局一批电影院；加强对新闻出版、文学艺术事业的引导和支持力度，抓好乡镇、街办文化站建设，丰富群众文化生活；深入开展“扫黄打非”工作，营造健康向上、文明和谐的文化环境。

6、积极促进体育发展。按照承办第七届全国城市运动会的要求，加快南昌国际体育中心、南昌水上运动中心的规划建设，大力培养体育人才，力争竞技体育在全国省会城市保持中上水平，广泛开展群众性体育活动和全民健身运动，迎接2008年奥运会和2011年全国城市运动会的召开。

7、认真贯彻基本国策。稳定低生育水平，保持人口适度规模及人口结构的平衡，促进人口素质提高。严格依法管理土地，推进节约集约用地，提高土地利用综合效益。以创建国家环保模范城为抓手，大力治理现有污染源，坚决抓好新上项目的环境影响评估和监控，切实保护生态环境。实施生态防护林体系工程和湿地保护及自然保护区建设工程，夯实我市生态建设基础。城市绿化覆盖率达到45%，森林覆盖率达到20.1%，城市集中式饮用水源水质力争达到二级以上标准，重点工业企业污染物达标排放，空气质量优良率、生活污水处理率进一步提高。

8、全力维护社会稳定。坚持预防为主、安全第一、综合治理，加强人民信访、人民调解、行政调解、安全生产、城乡消防、森林防火、食品药品监管等工作。有效实施“五五”普法和依法治市规划。着力加强社会治安综合治理工作，依法严厉打击刑事犯罪行为，全面推行社区和农村警务战略，加快保安服务业的发展，进一步完善社会治安防控体系，强化社区技防建设，基本建成覆盖全社会的技防网络，使南昌成为全省社会治安持续稳定的“首善之区”，不断增强人民群众的安全感。建立健全分类管理、分级负责、条块结合、属地为主的应急管理体制，有效应对自然灾害、事故灾难、公共卫生事件、社会安全事件，提高危机管理和抗风险能力。进一步增强国防观念，加强民兵预备役和人民防空建设，巩固“双拥”工作成果。高度重视基层社区（村）的基础作用，加强设施建设，完善服务网络，构建和完善政府扶持、社区（村）参与的机制，化解矛盾，提供服务，让百姓生活安康，社会稳定祥和。

三、进一步加强政府自身建设

刚刚胜利闭幕的市第九次党代会确定了南昌未来五年发展的总的要求和奋斗目标，实现这些要求和目标，任务光荣而艰巨。新一届政府一定要把思想和精力充分凝聚到实现“富民强市”目标、做好“两篇文章”上来，凝聚到实现“一个基地、三个中心、四个南昌”要求上来，坚持科学、民主行政和依法办事，进一步加强自身建设，不断提高行政效能，使我们的政府成为依法行政、诚实守信、廉洁高效、勤政为民的政府。

坚持科学发展，促进南昌快速和谐崛起。思路决定出路，观念就是财富。未来五年，既是发展的黄金期，也是矛盾的凸显期，改革、发展、稳定的任务非常繁重。我们要进一步解放思想，更新观念，开阔视野，自觉把南昌的发展置于全国乃至全球的发展格局之中去谋划。坚持把加快发展与科学发展有机结合起来，实现可持续发展，既要金山银山，更要绿水青山，创建资源节约型、环境友好型社会。要按照以发展求和谐、以和谐促发展的要求，在保持政策的稳定性、连续性的基础上，坚持与时俱进、开拓创新，不断推动经济社会又好又快地发展，实现南昌崛起的新跨越。

强化宗旨意识，切实维护人民群众的根本利益。坚持立党为公、执政为民。进一步拓宽渠道，倾听民声，了解民意，汇集民智。建立健全重大决策立项调研、集体决策、专家咨询、社会公示和听证等制度，全面推行民主决策和政务公开；完善公众参与、专家论证和政府决策相结合的科学民主决策机制。突出抓好与人民群众的利益密切相关的热点问题，敢于和善于破解经济社会发展中的重大难点问题，努力为民多办实事、好事，改善人民生活，促进社会公平。

严格依法行政，进一步提高行政效能。认真贯彻实施《行政许可法》和国务院《全面推进依法行政实施纲要》，加强政府立法工作，推行行政执法责任制，推进综合执法改革，强化行政执法监督。自觉接受人大及其常委会的依法监督和政协的民主监督，自觉接受群众的监督和舆论监督。进一步提高政府的公信力，以诚信政府推动诚信社会的建设。切实抓好“十一五”规划和重大决策的落实，明确层级责任，确保政令畅通。按照“经济调节、市场监管、社会管理、公共服务”政府职责要求，加快职能转变，简化办事程序，创新管理制度，完善“一站式”集中联合办公模式，进一步提高工作质量和效率。

加强执政能力建设，不断提高公务员队伍素质。城市间的竞争，首先是干部队伍素质的竞争，尤其是领导团队能力和水平的竞争。我们一定要加强学习，团结共事，凝聚力量，形成合力，把科学发展观的要求转化为实现富民强市、构建和谐社会的实际能力，不断提高审时度势的理解力、总揽全局的驾驭力、开拓进取的创新力、雷厉风行的执行力、攻坚克难的操作力。认真抓好《公务员法》的实施工作，加强公务员培训、交流和挂职锻炼工作力度，在实际工作中拓宽视野、增长才干。大力发扬艰苦奋斗的优良传统，严格执行领导干部廉洁从政各项规定，严格落实党风廉政建设责任制，积极推进惩治和预防腐败体系建设，从源头上预防和治理腐败。以纠正群众反映的突出问题为重点，加大纠风专项治理力度，强化公共资源招标投标监管，坚决查处违纪违法案件，为南昌又好又快发展营造一个风清气正的环境。

各位代表，过去的五年是南昌取得骄人业绩的五年，今后的五年，我们肩负着促进南昌快速崛起、经济跨越腾飞、全面实现小康社会的历史使命。让我们紧密团结在以胡锦涛同志为总书记的党中央周围，高举邓小平理论和“三个代表”重要思想伟大旗帜，全面落实科学发展观，在省委、省政府和市委的正确领导下，团结一心，锐意进取，善谋实干，奋发有为，共同创造南昌更加辉煌的明天！
